# Unraveling the Serum Protein Landscape in Celiac Disease: Current Evidence and Future Directions

**DOI:** 10.1002/iid3.70169

**Published:** 2025-05-05

**Authors:** Sajjad Bakhtiari, Behrooz Ahmadi, Nastaran Asri, Mostafa Rezaei‐Tavirani, Somayeh Jahani‐Sherafat, Andrea Masotti, Mohammad Rostami‐Nejad

**Affiliations:** ^1^ Gastroenterology and Liver Diseases Research Center, Research Institute for Gastroenterology and Liver Diseases Shahid Beheshti University of Medical Sciences Tehran Iran; ^2^ Celiac Disease and Gluten Related Disorders Research Center, Research Institute for Gastroenterology and Liver Diseases Shahid Beheshti University of Medical Sciences Tehran Iran; ^3^ Proteomics Research Center, Faculty of Paramedical Sciences Shahid Beheshti University of Medical Sciences Tehran Iran; ^4^ Laser Application in Medical Sciences Research Center Shahid Beheshti University of Medical Sciences Tehran Iran; ^5^ Bambino Gesù Children's Hospital‐IRCCS, Research Laboratories Rome Italy

**Keywords:** biomarkers, celiac disease, pathophysiology, serum proteins

## Abstract

**Background:**

Celiac disease (CD) is a chronic autoimmune disorder characterized by an abnormal immune response to gluten, leading to intestinal inflammation and various clinical manifestations. Serum proteins are increasingly recognized as potential biomarkers in CD, reflecting inflammation, malabsorption, and immune activation.

**Objective:**

This review aims to elucidate the role of serum proteins in the pathogenesis, diagnosis, and management of CD, emphasizing their potential as noninvasive biomarkers and therapeutic targets.

**Methods:**

A comprehensive review of current literature was conducted, focusing on key serum proteins such as albumin, transthyretin (TTR), transferrin, β2‐microglobulin (β2M), C‐reactive protein (CRP), and immunoglobulins. Their alterations in CD and their relevance to disease activity, nutritional status, and treatment response were examined.

**Results:**

CD‐related inflammation leads to increased acute‐phase proteins (e.g., CRP) and decreased transport proteins (e.g., albumin, TTR, transferrin), contributing to malnutrition and anemia. TTR serves as a sensitive marker of nutritional status, while transferrin levels correlate with iron deficiency, a common CD complication. Immunoglobulin profiles reflect immune responses to gluten. These proteins provide insights into CD pathophysiology and offer potential utility for diagnosis and monitoring.

**Conclusion:**

Serum proteins represent promising biomarkers for CD diagnosis and management, with potential for integration into clinical practice. Further research is necessary to validate their utility in routine patient care and explore their role in personalized therapeutic strategies.

## Graphical abstract

Celiac disease (CD) is a chronic autoimmune disorder characterized by an abnormal immune response to gluten, leading to intestinal inflammation and various clinical manifestations. Transthyretin (TTR) is notably sensitive to nutritional status, serving as a potential marker of malabsorption. Other serum proteins like transferrin and β2M have been investigated as biomarkers to monitor disease activity and treatment response. Acute‐phase proteins like C‐reactive protein (CRP) reflect inflammatory processes associated with the disease. Immunoglobulins also play a crucial role in the immune response to gluten proteins.

## 1 Introduction

Celiac disease (CD) is a chronic autoimmune intestinal disease caused by the unusual immune response to gluten, a group of proteins found in wheat, barley, and rye [1]. In recent decades, the global incidence of this disease has witnessed a rise, currently impacting around 1% of the worldwide population [2]. Genetic predisposition plays a central role in the pathogenesis of CD, with the strongest associations linked to human leukocyte antigen (HLA) Class II molecules, specifically HLA‐DQ2 and HLA‐DQ8 [3]. While ~90%–95% of CD patients carry the HLA‐DQ2 haplotype and most others carry HLA‐DQ8, these markers alone are not sufficient for disease development, as they are found in up to 30%–40% of the general population without CD. This indicates that additional environmental and immunological factors are critical contributors to disease onset [4].

Gluten initiates the disruption of interenterocyte tight junctions (TJs) through its interaction with intestinal epithelial cells. This disruption results in increased intestinal permeability, which is mediated by elevated levels of zonulin, a peptide that plays a critical role in the regulation of TJ functionality [5]. As a result, gliadin peptides pass through the epithelial barrier and activate T cells in the lamina propria. Upon activation, these T cells release a significant amount of cytokines that promote inflammation [6]. This inflammatory process leads to mucosal inflammation, malabsorption, and disruption in the balance of essential nutrients [7, 8]. CD manifests with a broad spectrum of symptoms, ranging from mild gastrointestinal discomfort to severe malabsorption syndromes. The condition is also associated with extraintestinal manifestations and long‐term complications, including weight loss, growth failure, and an increased prevalence of other autoimmune diseases [9, 10, 11].

Recent advancements in multiomics approaches have opened new avenues for understanding CD at a molecular level. By integrating data from genomics, proteomics, and metabolomics, researchers can gain a holistic view of the disease pathogenesis and its complex interactions within the biological system. This integrative methodology allows for a more comprehensive characterization of the serum protein landscape in CD, facilitating the identification of potential biomarkers that may not be evident when examining each omics layer independently. Such technologies can illuminate the dynamic changes in metabolic profiles and immune responses in CD patients [12, 13].

Serum contains numerous proteins with distinct functions. These proteins are essential for maintaining blood pressure, regulating plasma pH, and transporting essential nutrients. Furthermore, serum proteins are essential in the immune response to gluten and contribute significantly to the inflammation observed in CD. Among these proteins, cytokines serve as key signaling molecules involved in cell communication and have been shown to be associated with the pathogenesis of CD [14, 15]. For instance, interleukin (IL)‐15 has been shown to play a role in the activation of intraepithelial lymphocytes (IELs), which are involved in the immune response against gluten in celiac patients [16]. Furthermore, the levels of certain immunoglobulins (Ig), such as IgA, are altered in CD patients, affecting the immune response and contributing to the CD pathogenesis [17, 18]. Noteworthy is the crucial involvement of serum proteins in the inflammatory response associated with CD, as alterations in their levels can influence the progression of the disease [19]. For example, acute‐phase proteins such as C‐reactive protein (CRP) are elevated in response to inflammation and tissue damage associated with the disease [20].

Despite the advancements in our understanding of CD and the management strategies available, significant gaps remain in the current diagnostic and therapeutic landscape [16]. Current diagnostic and therapeutic approaches in CD heavily rely on serum protein biomarkers such as anti‐tissue transglutaminase (tTG) and anti‐deamidated gliadin peptide (DGP) antibodies, which play a crucial role in diagnosing the disease and monitoring adherence to gluten‐free diets. Alterations in the composition of other serum proteins may serve as valuable tools for the clinical assessment of patients suspected of having CD, thereby facilitating early detection and ongoing monitoring of disease progression. Additionally, a comprehensive understanding of the specific interactions between serum proteins and gluten peptides in the context of CD can provide insights into the disease underlying mechanisms [21]. Consequently, this review aims to investigate the current literature regarding the interactions between serum proteins and CD. Although numerous proteins in serum perform a variety of functions, this review will focus on the most significant serum proteins implicated in CD, exploring their multifaceted roles in the disease development, diagnosis, progression, and treatment, and highlighting their importance within this complex condition.

**Figure 1:**
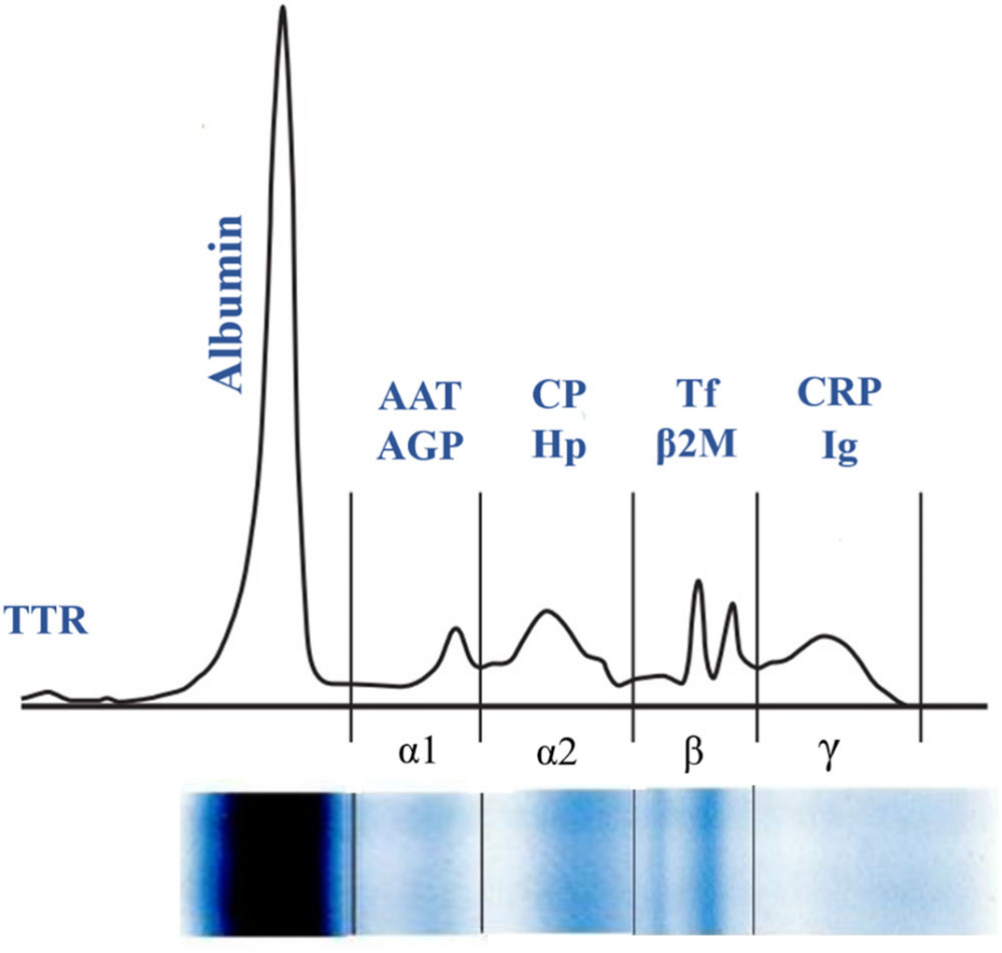
Classification of investigated proteins in celiac disease based on serum protein electrophoresis. TTR, transthyretin; AAT, alpha‐1 antitrypsin; AGP, alpha‐1 acid glycoprotein; β2M, beta‐2‐microglobulin; CP, ceruloplasmin; CRP, C‐reactive protein; Hp, haptoglobin; Ig, immunoglobulins; Tf, transferrin [24].

Serum proteins constitute a complex mixture of proteins that are essential for numerous physiological processes, including the maintenance of osmotic pressure, molecular transport, immune response modulation, and enzymatic activity [22]. The concentration of total serum protein serves as an indicator of protein loss associated with hepatic and renal dysfunction. Furthermore, assessing total serum protein helps evaluate the body's nutritional condition and aids in diagnosing specific diseases. Various methods, including the Kjeldahl, biuret, Lowry, Bradford, and UV absorbance, have been established to assess serum protein levels [23]. Serum protein electrophoresis (SPE) is an easy‐to‐perform test used to separate serum proteins according to their size and electrical charge, helping identify conditions like inflammation, malignancies, liver or kidney failures, and hereditary protein disorders. By this method, serum proteins can be categorized into two primary groups: albumins and globulins. The most prominent peak corresponds to albumin, which is situated near the positive electrode. In contrast, globulins—comprising alpha1, alpha2, beta1, beta2, and gamma fractions—exhibit their peaks closer to the negative electrode, with the gamma globulin peak located nearest to it [24]. Figure 1 demonstrates a typical normal distribution of proteins through SPE.

The body's responses to inflammation, infection, and injury are collectively called acute‐phase response, which encompasses both local and systemic effects. A significant aspect of this response is the alteration in the levels of serum proteins, primarily synthesized in the liver, known as acute‐phase proteins (APPs) [25]. Aside from liver production, these proteins are synthesized extrahepatically by epithelial, endothelial, and connective tissue cells. α1‐antitrypsin, α1‐antichymotrypsin, α1‐acid glycoprotein, ceruloplasmin, fibrinogen, plasminogen, and haptoglobin are among the proteins that show an increase during acute phase response while albumin, transthyretin, and transferrin concentration reduce during this phase [26].

The essential role of APPs involves stimulating the complement system, binding cellular remnants such as nuclear components, neutralizing enzymes, scavenging free radicals, and regulating the immune response of the host [27]. The peak serum levels of APPs typically occur within 24–48 h following the onset of inflammation. A decline in APP concentrations is indicative of the patient's recovery from the inflammatory process. In cases of chronic inflammation, elevated levels of APPs are frequently observed; however, these levels are generally lower than those seen during acute inflammatory episodes [28].

APPs are triggered by the release of cytokines like IL‐1, IL‐6, and tumor necrosis factor (TNF)‐α from macrophages (MQs) and monocytes at inflammatory sites. TNF‐α, IL‐1, and interferon (IFN)‐γ are vital for the expression of inflammatory mediators such as prostaglandins and leukotrienes, which induce the production of platelet‐activating factor and IL‐6. Following stimulation by proinflammatory cytokines, Kuffer cells in the liver produce IL‐6 and present it to hepatocytes. IL‐6 serves as the major mediator responsible for the hepatocytic secretion of most APPs.

**Figure 2:**
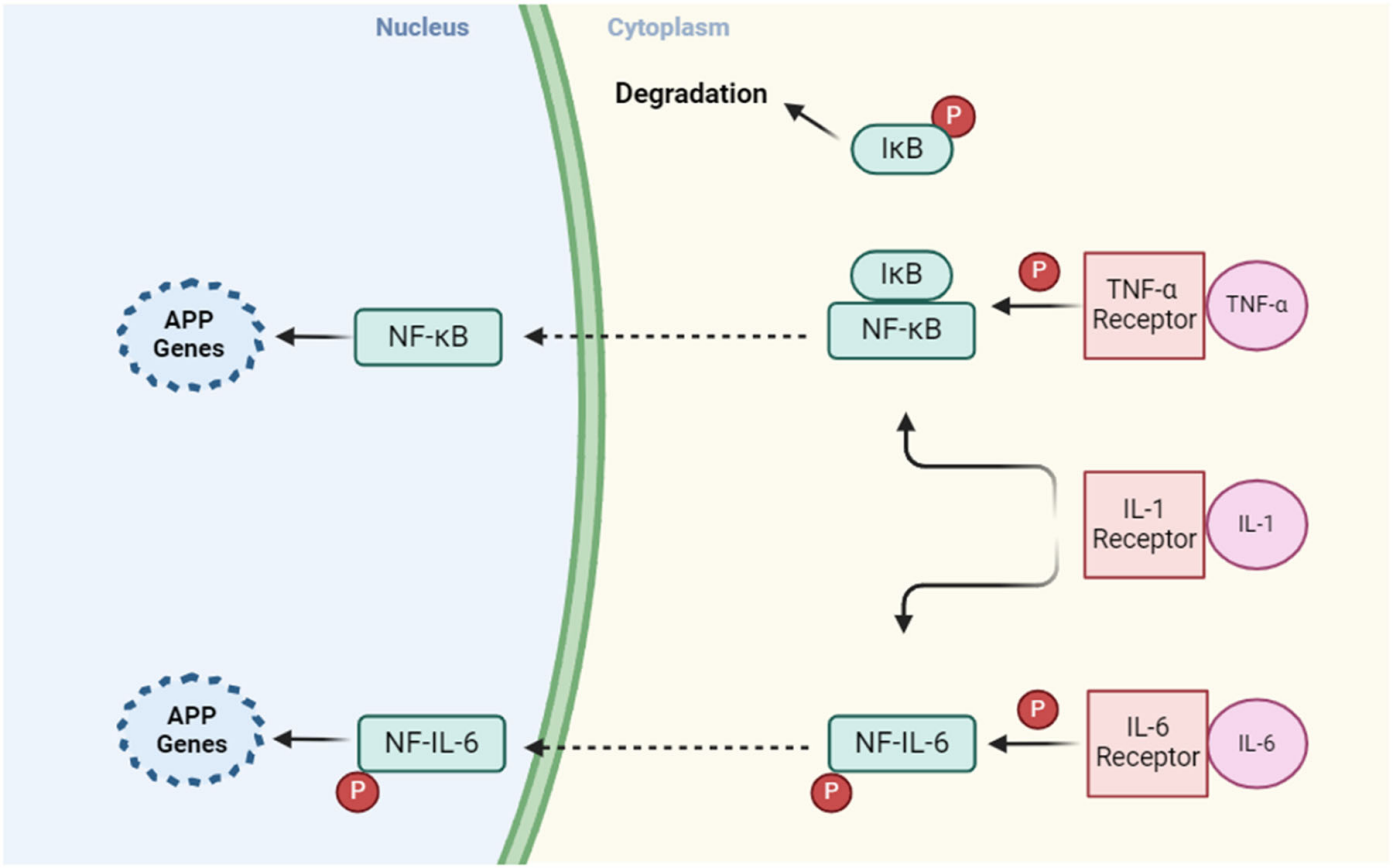
Signaling pathways that mediate the expression of acute phase proteins (APPs). The binding of TNF‐a and IL‐1 to their receptors leads to the activation of IKK, which then phosphorylates IκB, an inhibitor of NF‐κB in the cytoplasm. The degradation of phosphorylated IκB occurs through the ubiquitin–proteasome pathway, resulting in the liberation of NF‐κB. Subsequently, NF‐κB migrates to the nucleus to stimulate the transcription of genes associated with APPs. IL‐6 binds to its receptor, leading to NF‐IL6's phosphorylation. This phosphorylated NF‐IL6 then moves to the nucleus, stimulating the transcription of genes related to acute phases. The activation of the IL‐1β receptor can also cause NF‐IL6's phosphorylation.

An alternative proposed mechanism of inflammation associated with APPs involves the activation of the nuclear factor kappa‐light‐chain‐enhancer of activated B cells (NF‐κB) signaling pathway. Upon binding of TNF‐α and IL‐1β to their receptors, the IκB kinase gets activated. This results in the phosphorylation of IκB, an inhibitor associated with NF‐κB in the cytoplasm. Consequently, the phosphorylated IκB is degraded through the ubiquitin–proteasome pathway, allowing the free NF‐κB to migrate to the nucleus and promote the transcription of acute‐phase genes [26, 27]. The signaling pathways of APPs are illustrated in Figure 2.

## 2 Transthyretin

Transthyretin (TTR) is a homotetrameric protein, primarily produced by the liver and choroid plexuses of the brain. It is also synthesized in other tissues, including the retinal pigment epithelium, pancreatic islet cells, and to a lesser extent, the heart, spleen, and skeletal muscle [29]. TTR has multiple functions, including transporting thyroid hormones and retinol as a carrier, exhibiting proteolytic activity on several substrates, affecting the nervous system in various ways, such as behavior and cognition, and protecting against ischemic effects and Alzheimer's disease [30]. TTR has a short half‐life in the blood, typically 2–3 days. This protein is sensitive to changes in protein‐energy status and reflects recent dietary intake, therefore, can be used to assess malnutrition. However, TTR levels can drop rapidly during an acute‐phase response, which makes it challenging to interpret plasma TTR levels in patients with infections, inflammation, or recent trauma [31, 32]. TTR as an inflammatory protein, may play a role in the pathophysiology of CD by contributing to malnutrition associated with impaired nutrient absorption. However, the relationship between TTR and the inflammatory response in CD is not well characterized, and current literature is limited.

In 1998, a pioneering study conducted by Reifen and colleagues examined the changes in TTR as a marker of disease activity in children with CD in comparison with healthy controls. They found that untreated CD patients had significantly lower levels of TTR compared to healthy children and patients under treatment with a GFD [33]. Three years later, McMillan and colleagues examined changes in TTR levels in patients with CD as a potential indicator of mucosal recovery. Since malnutrition is a common issue in individuals with CD, the researchers measured TTR levels before and after 12 months on a GFD to assess their correlation with duodenal histology. They found that TTR levels increased in patients who experienced mucosal improvement. In contrast, TTR levels decreased in those who did not show improvement and the levels of this protein did not correlate with the degree of villous atrophy. The study concluded that serial measurements of TTR can be used as a noninvasive way to track mucosal recovery in CD patients, potentially reducing the need for follow‐up biopsies and their frequency [34]. While inflammation and malnutrition are potential pathways linking TTR to CD, further investigation is required to elucidate the mechanisms underlying this association.

## 3 Albumin

Albumin is the predominant protein in plasma, characterized as a complex macromolecule that is synthesized by hepatocytes and promptly released into the bloodstream. This protein accounts for approximately half of the total protein present in the plasma in healthy individuals [35]. Serum human albumin plays a crucial role in regulating plasma oncotic pressure and serves as a carrier for various substances, including bilirubin, ions, fatty acids, and exogenous compounds like drugs [36]. In clinical practice, hypoalbuminemia is often associated with a range of underlying conditions, including inflammation, liver diseases, protein‐losing enteropathy, nephrotic syndrome, trauma, and cancer [37].

Several studies have investigated serum albumin in CD. Poorly managed CD can lead to the intestinal mucous membrane flattening, resulting in the loss of significant amounts of protein and malabsorption of essential nutrients. This can trigger chronic immune system activation and hypoalbuminemia [38]. Siddiqui and colleagues examined the albumin imbalance in children newly diagnosed with CD and assessed the necessity of comprehensive metabolic evaluation at diagnosis. Their study found hypoalbuminemia in all CD patients, underlining the importance of thorough metabolic assessment, particularly in cases of severe malnutrition [19].

In a study conducted by Doganci and Bozkurt, the researchers investigated serum albumin levels in children diagnosed with CD, categorizing the participants into three distinct groups based on their clinical presentations including those exhibiting chronic diarrhea, those with iron deficiency, and those with short stature. The findings indicated that the mean serum albumin level was lower in the group suffering from chronic diarrhea compared to the other two groups; however, this difference did not reach statistical significance [39].

Studies on patients on GFD also showed similar results, as Masood and Ali Shaikh analyzed the biochemical profiles of adult patients with CD and found that patients had lower levels of serum albumin compared to healthy controls [40]. However, research indicates that serum albumin levels normalize following the implementation of a GFD, as noted by Meena and colleagues in their report. They report a 56‐year‐old male patient presented with a 3‐month duration of pedal edema, abdominal distension, and loss of appetite. Hypoalbuminemia was the only abnormal laboratory test. After ruling out renal, cardiac, and hepatic causes of hypoalbuminemia and edema, a duodenal biopsy was performed, which showed partial villous atrophy with increased IELs and crypt hyperplasia. Additionally, tTG antibody levels were elevated in the serum leading to the diagnosis of CD. After 3 months of adherence to GFD, the albumin returned to its normal level [41]. The duration for returning the albumin level to a normal value varies among studies and requires further investigations to elucidate the cause. A similar observation was made in the Repo and colleagues study while the median time of adhesion to GFD was 7 months [42]. Essrani and Berger investigated the effect of GFD on CD patients with chronic liver disease. The researchers tracked changes in serum albumin levels from diagnosis to 6 months after starting the diet. The study found that the average albumin level remained stable during this period. However, the investigators attributed this finding to the study's limited sample size [43].

Hypoalbuminemia was also reported in severe and acute forms of CD, which is characterized by severe diarrhea, dehydration, and metabolic disturbances. This condition can lead to various electrolyte imbalances, including hypokalemia, hyponatremia, hypocalcemia, hypomagnesemia, and hypoalbuminemia [44].

Albumin levels were considered important for staging refractory celiac disease (RCD). This condition is characterized by persistent symptoms, severe malabsorption, and intestinal damage despite adherence to a strict GFD. Rubio‐Tapia and colleagues have identified five factors that predict mortality in patients with RCD, with low albumin levels (< 3.2 g/dL) being the most significant predictor when present at the time of diagnosis [45].

While direct studies specifically linking serum albumin to CD severity are limited, emerging evidence from related gastrointestinal conditions indicates that lower albumin levels often reflect increased disease activity. For instance, in Crohn's disease, serum albumin levels have been found to inversely correlate with disease activity [46]. Given its association with malnutrition and nutritional absorption, hypoalbuminemia can serve as an indirect marker of the extent of intestinal damage and absorptive capacity [47]. Furthermore, when assessing albumin as a nutritional marker, it is essential to consider its variability during acute‐phase responses, which can complicate interpretations [48]. Therefore, a comprehensive evaluation that combines serum albumin measurements with clinical assessments, dietary evaluations, and inflammatory markers may provide a more accurate reflection of a patient's nutrition, disease progression, and overall health status in CD.

In conditions of hypoxia, acidosis, and oxidative stress, the interaction of specific metals with the N‐terminal region of albumin is impaired, resulting in the formation of ischemia‐modified albumin (IMA) [49]. Recently, IMA has been used to indirectly measure the presence of oxidative stress and ischemia [7]. The importance of IMA in CD is highlighted by the research studies conducted by Yuksel et al. and Sayar et al. [50, 51]. Yuksel and colleagues found that patients with CD had substantially higher IMA levels compared to healthy controls. In patients with CD, the high levels of IMA may be a consequence of tissue damage in the gastrointestinal tract. Furthermore, the increased inflammation and autoimmune response that occurs in the disease are also accompanied by oxidative stress [52]. Another study conducted by Sayar and colleagues found that pediatric patients with CD exhibited elevated levels of IMA at the time of diagnosis when contrasted with a control group. Furthermore, after the implementation of a GFD, IMA levels in these patients significantly decreased in comparison to the control group. They also detected a correlation between IMA and the histopathological stage [51]. In summary, researchers concluded that IMA levels may serve as a diagnostic and monitoring tool for evaluating treatment effects in active CD by comparing pre‐ and posttreatment measurements. Given the significance of oxidative stress in CD, IMA could serve as a biomarker for oxidative stress, which is likely to decrease after GFD treatment [50, 51].

The measurement of serum albumin levels could aid in the diagnosis of CD, particularly in cases where symptoms are mild or atypical. In addition to diagnosis, serum albumin measurement may also be useful in monitoring treatment response and disease progression as following the initiation of a GFD, a significant increase in serum albumin levels is often observed [44].

## 4 Alpha‐1 Antitrypsin

Alpha‐1‐antitrypsin (AAT) is an acute‐phase protein belonging to the serine protease inhibitor (SERPIN) superfamily. AAT plays a crucial role in protecting tissues from enzymes released during inflammation, such as neutrophil elastase and proteinase 3 [53]. The AAT gene has common alleles, including M, S, Z, and null. The M allele is considered normal, while the S and Z alleles are associated with AAT deficiency (AATD). The null allele does not express any AAT protein in the blood [54]. In a study involving 111 patients with CD, researchers found that 95 of them exhibited the MM phenotype [55].

The study by Walker‐Smith and Andrews in 1972 was one of the first to investigate the relationship between CD and AATD. The researchers found that among 13 untreated children with CD, 4 had low levels of AAT. Moreover, in a separate group of 15 children with CD who were following a GFD, 5 children also had low levels of AAT [56]. Years later, Pons Romero and colleagues reported a case of a 50‐year‐old woman with AATD (PiSZ phenotype) who developed CD, suggesting a potential association between AATD and CD [57]. Blanco and colleagues found that serum AAT levels increased in CD patients, although this increase was not statistically significant [58]. In contrast, Klasen and colleagues discovered no genetic association between AAT and CD suggesting that there is no connection between them [59]. This inconsistency highlights the need for further research to clarify the connection between AAT and CD.

## 5 Alpha‐1 Acid Glycoprotein

Alpha‐1 acid glycoprotein (AGP), or orosomucoid, is an abundant serum glycoprotein produced by hepatocytes involved in pharmacokinetics and immune modulation. The concentration of AGP can increase significantly, by as much as fivefold, during inflammatory events, establishing it as a key member of the acute‐phase protein family [60]. The association between serum AGP and CD is a relatively underexplored area of research. Despite its established role in inflammatory processes, only one study has investigated the link between AGP levels and CD. In this study, Blanco and colleagues found that AGP levels increased in patients with CD, however, this increase was not statistically significant [61].

## 6 Ceruloplasmin

The glycoprotein ceruloplasmin (CP), synthesized by hepatocytes, is a key component of the blood that carries 40%–70% of copper. It is involved in various physiological processes, including facilitating copper transport, regulating iron levels, and participating in antioxidant and free radical‐scavenging activities. This protein also catalyzes the oxidation of multiple substances, including copper, iron, and other organic compounds [62]. The level of this acute‐phase protein increases in response to infections, cancers, cholangitis, and copper toxicity. In contrast, the CP level decreases in cases of liver disease, malnutrition, malabsorption, and nephrotic syndrome [63].

Previous research has yielded conflicting findings regarding the prevalence of copper deficiency in individuals with CD, with some studies indicating a possible link and others failing to detect a significant correlation. Probable copper deficiency in CD patients is thought to be related to intestinal malabsorption [64, 65]. The measurement of CP levels in individuals with CD may be useful in identifying those who are at risk of copper deficiency. A study by Botero‐López and colleagues investigated the connection between copper deficiency and CD by examining CP levels in patients with CD compared to healthy controls. The research revealed that 15% of patients suffered from copper deficiency, while 10.9% had decreased CP levels [66]. Similarly, Cavallieri and colleagues reported a case of myelopathy induced by copper deficiency secondary to undiagnosed CD, attributing low serum CP levels to impaired copper absorption [67]. In contrast, Ince and colleagues found no significant difference in CP levels between CD patients and the control group [68]. However, a case study by Roumeliotis and colleagues reported a normal CP level in a 16‐year‐old girl who was diagnosed with CD and liver cirrhosis of unknown etiology [69].

These conflicting findings suggest that CP measurement may not be a reliable indicator of copper deficiency in all cases of CD, as some studies did not detect significant differences in CP levels between patients and controls. Additionally, copper deficiency may not be a universal feature of CD, with varying results across studies. Furthermore, CP is an acute‐phase protein that can increase in response to inflammatory states, which may confound the diagnosis of copper deficiency. Therefore, the diagnosis of copper deficiency in CD patients may require additional tests or biomarkers beyond CP measurement, and further research is needed to clarify the role of CP measurement in CD.

## 7 Haptoglobin

Haptoglobin (Hp) is a tetrameric glycoprotein produced in multiple tissues and cell types. This acute‐phase protein binds to hemoglobin (Hb), preventing excessive oxidative stress and transporting it to MQs, where it is safely removed from the bloodstream as a Hp–Hb complex [70]. Additionally, it has been demonstrated that Hp binds to a substantial majority of CD4+ and CD8+ T lymphocytes, thereby directly inhibiting their proliferation and affecting the balance between T helper (Th) 1 and Th2 cell populations [71]. Genetic variations in the Hp gene or associated genes may play a role in an individual's susceptibility to CD and may also influence the disease progression [71].

The human Hp gene has two distinct variants, Hp1 and Hp2, which differ only in the alpha chain. As individuals have two copies of the gene, one inherited from each parent, this results in three possible genetic combinations Hp1‐1, Hp1‐2, and Hp2‐2 [72]. The antioxidant, scavenging, and immunoregulatory functions of Hp vary depending on the individual's phenotype. In addition, genetic variations in Hp have been found to impact the progression of various inflammatory and autoimmune disorders [73]. In 2008, Papp and colleagues conducted the first study on the relationship between Hp polymorphism and symptoms in CD patients. The study found that CD patients exhibited a significantly higher prevalence of the Hp2‐1 phenotype in comparison to the general population. Conversely, individuals with the Hp2‐2 phenotype demonstrated a lower prevalence; however, they were more prone to severe malabsorption and failure to thrive [74].

A study by Leonard and colleagues found that the distribution of Hp genotypes was not associated with the risk of developing CD autoimmunity in patients with type 1 diabetes (T1DM), who were analyzed for the frequency of CD autoimmunity and distribution of Hp genotypes [75].

Despite the established importance of Hp polymorphism in autoimmune disorders, the understanding of its specific role in CD is limited. As a result, the mechanisms by which Hp polymorphism influences CD development and progression remain poorly understood. Further research is needed to elucidate the complex interplay between Hp and CD.

## 8 Transferrin

Iron deficiency anemia (IDA) is a common manifestation of CD, occurring in children and adults, particularly in patients with subclinical or atypical forms of CD [76]. The prevalence of anemia in newly diagnosed CD patients ranges widely, from 12% to as high as 85%, according to different studies [77, 78]. Studies have shown that CD patients presenting with anemia may exhibit more severe disease manifestations than those with diarrhea. Furthermore, anemic patients have a longer duration of illness and a more severe presentation of serological and histological markers at diagnosis [79].

Transferrins (Tf) are a family of proteins that play a crucial role in maintaining iron homeostasis. They bind to free iron in the blood and body fluids, using a receptor‐mediated process to deliver iron to cells while removing toxic free iron from the circulation [80]. Tf level testing is a diagnostic tool used to uncover the underlying cause of anemia, analyze iron metabolism, and measure the blood's ability to carry iron. Elevated Tf levels typically indicate low iron status, as a reduction in iron bound to Tf results in higher concentrations of unbound Tf. This may serve as an indicator of IDA [81]. The significance of Tf in IDA has prompted several studies to evaluate Tf levels in CD patients. Siddiqui and colleagues examined the hematological manifestations and Tf levels of untreated children with CD. The results revealed a significant increase in Tf levels among these patients compared to healthy controls [82]. This finding was consistent with his previous research in adults, which also observed a significant increase in Tf levels among untreated adult CD patients compared to healthy controls. The findings suggest that measuring Tf levels in serum could be a useful diagnostic tool for CD, enabling effective treatment and symptom alleviation [38]. The increase of Tf in CD patients was also observed in the Kårhus and colleagues study, which investigated possible biochemical abnormalities associated with CD [83]. In another study, Gubska and colleagues investigated the serum levels of Tf in adult CD patients who were following a GFD. The study found that the average Tf levels in the patient group were comparable to reference values, suggesting that prolonged adherence to a GFD may facilitate the restoration of the normal structure of the intestinal mucosa [84]. Research has shown that individuals with Marsh IIIa, IIIb, and IIIc (a classification system used to categorize the degree of small intestinal mucosal damage in CD patients), particularly those with Marsh IIIc, tend to have significantly lower iron levels compared to those with Marsh 0, I, or those with milder mucosal damage [85]. In this regard, to explore the potential of Tf as a biomarker for monitoring CD progression and iron storage, Hasan and colleagues analyzed the serum Tf levels of adult patients with CD at various stages of histological severity. They found that CD patients have higher levels of Tf compared to control groups, which is consistent with other research. However, the study did not detect any significant differences in Tf concentration between patients with Marsh IIIa, b, and c and those with Marsh 0 and I. The researchers concluded that serum Tf levels are not a suitable marker for tracking disease progression [86].

The conventional serum Tf measurement is often inadequate for diagnosing and managing iron deficiency in CD patients due to its susceptibility to inflammation‐induced variability in chronic diseases. This limitation underscores the need for more accurate and comprehensive diagnostic tools. The use of a single test may not be sufficient to rule out IDA, as each test has its limitations and a combination of tests such as serum iron, ferritin levels, soluble transferrin receptor (sTfR), Tf saturation, total iron‐binding capacity, and red blood cell indices should be employed to accurately diagnose and monitor IDA in CD patients [87, 88].

## 9 Beta‐2‐Microglobulin

Beta‐2‐microglobulin (β2M) is a crucial component of the major histocompatibility complex Class I (MHC‐I) molecule, which is expressed on the surface of nearly all nucleated cells. This protein plays a vital role in facilitating the presentation and processing of antigens, modulating the inflammatory response, and influencing the complement cascade and stress response [89]. β2M is consistently released from cells into the bloodstream, and under normal circumstances, it is primarily eliminated by the kidneys. Several studies have focused on β2M as a biomarker for autoimmune disorders such as systemic lupus erythematosus, Sjögren's syndrome, and its potential association with inflammatory bowel disease and kidney function [90, 91, 92, 93].

The examination of β2M in CD has a historical foundation, originating from the pioneering study conducted by Blanco and colleagues in 1985, who were the first to evaluate serum concentrations of β2M in pediatric patients with CD. Their findings indicated significantly elevated levels of this protein in patients, which may be attributed to intestinal infiltration or lymphocyte activation. This elevation has the potential to serve as a noninvasive biomarker for monitoring treatment response and assessing the activity of CD [58]. Five years later, Bonamico et al. [94] investigated this protein in CD patients who had been on a GFD. They discovered that the serum β2M level of patients on a GFD for < 8 months was significantly different from those on a GFD for more than 8 months and those from the control group. Additionally, no significant difference was found between patients on a GFD for more than 8 months and the control group. Hence, serum β2M levels in patients with CD may serve as a biomarker for evaluating the effectiveness of a GFD. The significance of β2M in monitoring the effectiveness of treatment with a GFD was also investigated in a study by Kochańska‐Dziurowicz and Bukowska, where they examined serum levels of this protein in children with CD. The study discovered that in children with an established diagnosis of CD, the levels of β2M were significantly higher compared to those who were on a GFD, and quantification of β2M concentrations in serum specimens of children with CD may serve as a valuable biomarker for monitoring the efficacy of GFD in managing the disease [95].

While β2M holds significant promise as a noninvasive biomarker for monitoring treatment response and assessing disease activity in CD, its clinical application is limited by several challenges. Elevated β2M levels lack specificity to CD, as they are also associated with conditions such as kidney dysfunction, malignancies, and other autoimmune diseases [96]. Additionally, non‐disease‐specific factors, including age, renal function, and concurrent infections, can influence β2M levels, complicating its use as a standalone biomarker [97]. To overcome these limitations, future research should aim to define disease‐specific thresholds and investigate the synergistic potential of combining β2M with other biomarkers to improve diagnostic accuracy. Furthermore, comparisons with established biomarkers, such as tTG antibodies, are essential to evaluate their relative feasibility and clinical utility in practice.

## 10 C‐Reactive Protein

CRP is an acute‐phase protein produced by the liver in response to releasing inflammatory cytokines. CRP concentrations typically rise 4–6 h after the onset of acute tissue injury or inflammation, and then rapidly decline as the inflammation resolves [98]. Due to their limited sensitivity, traditional CRP assays could not detect low levels of the protein in serum. To address this limitation, high‐sensitive assays (hs‐CRP) were developed and have since become a standard tool in clinical laboratories [99]. Measurement of CRP is a valuable tool in clinical practice, helping diagnose and track the progress of various acute and chronic inflammatory conditions such as cardiovascular, rheumatologic, infectious, and gastrointestinal diseases [100].

Research has highlighted the importance of CRP in CD, with several studies examining its role in this condition. In this regard, Cakir and Dogan conducted a comparative analysis of hs‐CRP levels in patients with CD and healthy controls, finding a statistically significant difference between the groups. In addition, they discovered a significant correlation between the systemic immune inflammation index, a novel inflammatory biomarker based on platelet, neutrophil, and lymphocyte counts, and hs‐CRP levels [101].

In another study, Pitocco and colleagues compared the levels of hs‐CRP among patients with CD, patients with T1DM, and patients with both conditions. They discovered that patients with T1DM and CD had higher levels of hs‐CRP than those with only one of these conditions. However, no significant differences were found between the three groups. According to the researchers, this might be attributed to the patient's metabolic and nutritional management, as all T1DM patients were receiving insulin therapy, and all CD patients were adhering to a GFD [102].

Several studies have shown that CD is linked to a higher risk of cardiovascular disease (CVD) and mortality, highlighting the need for early detection and intervention [103, 104, 105]. The discovery of potential biomarkers could play a crucial role in identifying individuals at high risk, enabling targeted prevention and treatment strategies. In this regard, Tetzlaff and colleagues assessed biochemical markers in newly diagnosed adult CD patients to identify risk factors and potential biomarkers for CVD. Their analysis revealed that these patients had higher levels of hs‐CRP compared to controls [20]. Caliskan and colleagues investigated the impact of CD on coronary microvascular circulation and explored the connection between coronary flow velocity reserve (CFVR) and the hs‐CRP/albumin ratio as a potential predictor of illness and malignancies. The CFVR ratio compares the blood flow to the heart muscle when it is under stress to when it is at rest. This ratio provides a comprehensive assessment of macrovascular and microvascular ischemia [106]. Researchers found that individuals with CD had significantly lower serum albumin levels and higher levels of hs‐CRP compared to healthy individuals. The hs‐CRP/albumin ratio was also significantly elevated in the CD group. In addition, the study revealed that hs‐CRP and hs‐CRP/albumin ratio were associated with low CFVR and there is a close relationship between subclinical atherosclerosis markers such as CFVR and hs‐CRP. The researchers concluded that CFVR is a practical and repeatable way to evaluate coronary microvasculature. This method, combined with inflammatory markers such as the hs‐CRP/albumin ratio, can help identify CD patients at risk of developing atherosclerosis. Furthermore, an increased hs‐CRP/albumin ratio may be a more effective indicator of coronary microvascular dysfunction in patients with CD than using hs‐CRP and albumin levels alone [107]. The increase in CRP levels was also observed in adult CD patients on a GFD in the study of Korkmaz and colleagues. The study investigated arterial stiffness as an independent predictor of CVD in adult CD patients without conventional cardiovascular risk factors. The results suggested that elevated arterial stiffness and CRP levels may be indicative of a potential link between inflammation and arterial stiffening [108]. A study by De Marchi and colleagues discovered that patients who strictly adhered to a GFD for 6–8 months experienced a decrease in CRP levels in comparison with the baseline [109].

While CRP levels are frequently elevated in patients with CD, it is essential to acknowledge the limitations associated with this biomarker. CRP is a dependable marker characterized by stable levels throughout the day, being unaffected by food intake, and possessing a prolonged duration and a wide dynamic range. However, elevated CRP levels can occur in various conditions unrelated to CD. Additionally, other factors such as age, body mass index, and smoking habits can also influence CRP levels, further reducing its specificity for diagnosing particular diseases [110]. Therefore, it is important to consider a comprehensive range of factors when evaluating CRP levels to ensure accurate diagnoses.

## 11 Immunoglobulins

Igs are composed of two heavy and two light chains, forming a heterodimeric structure. These proteins can be categorized into two main parts, the variable region, which is responsible for binding to specific antigens, and the constant region, which determines the protein's function. The five main classes of heavy chain constant domains give rise to distinct immunoglobulin isotypes, namely, IgM, IgG, IgA, IgD, and IgE, each with its unique characteristics [111].

The tTG‐IgA assay is widely regarded as the initial diagnostic test for CD and is also used to assess adherence to a GFD. However, IgA deficiency can result in false‐negative test outcomes [112, 113]. Selective immunoglobulin A deficiency (SIgAD) is recognized as the most prevalent primary immunodeficiency, characterized by undetectable serum IgA levels while maintaining normal serum IgG and IgM levels in patients over 4 years old, after excluding other causes of hypogammaglobulinemia. Partial IgA deficiency (PIgAD) is defined by serum IgA levels that are detectable but fall two standard deviations below the normal range, with IgG and IgM levels still within the normal range [114]. SIgAD is estimated to impact 2%–3% of patients with CD, which is ~10–15 times more common than in the general population. This correlation suggests that patients with SIgAD are at a higher risk for developing CD [113]. Chow and colleagues assessed the prevalence of selective and PIgAD in adults and children with CD and examined their serological response to a GFD. They found that selective and PIgAD was present in 2% of CD patients, with the condition being equally common in both adults and children [115].

The connection between IgA deficiency and CD extends to shared genetic predispositions. Both conditions are strongly linked to the HLA‐DQ2 and HLA‐DQ8 haplotypes, which influence immune tolerance mechanisms and predispose individuals to autoimmune disorders. This shared genetic foundation emphasizes the need to consider IgA levels in CD diagnosis, especially in patients with atypical presentations or false‐negative serology results [116].

SIgA regulates the gut microbiome by binding microbial antigens, preventing pathogen translocation, and promoting microbial balance. In CD, impaired IgA‐mediated responses can lead to dysbiosis, characterized by an imbalance in the gut flora. This dysbiosis exacerbates intestinal inflammation, compromises gut barrier integrity, and amplifies immune activation against gluten peptides [117].

In summary, IgA homeostasis is central to the immune response in CD, influencing both disease pathogenesis and diagnostic approaches

Considering the importance of IgA‐based assays in diagnosing CD, it is crucial to measure total IgA to ensure adequate levels. If IgA levels are undetectable, incorporating IgG‐based tests can help reduce the likelihood of false negative results [112]. Kumar and colleagues explored the effectiveness of IgG‐based tests for diagnosing CD in IgA‐deficient patients. They studied 15 IgA‐deficient patients with CD and 10 IgA‐deficient patients without CD. All 15 CD patients were positive for IgG EMA and IgG gliadin antibodies, and all but one also tested positive for IgG tTG antibodies. The study suggests that IgG‐specific tests for endomysium, gliadin, and tTG antibodies are effective in identifying IgA‐deficient patients with CD [113]. In another investigation, Pallav and colleagues assessed the prevalence of IgA deficiency by reviewing 1000 consecutive patients undergoing tTG‐IgA testing and 243 healthy controls. They found that the prevalence of SIgAD was slightly higher in CD patients compared to healthy controls, while the prevalence of PIgAD was similar between the two groups. The study indicates that IgG‐based testing is effective for detecting CD in patients with SIgAD [114]. Villalta and colleagues investigated whether IgG antibodies against deamidated gliadin (IgG‐anti‐DGP) could effectively diagnose CD in patients with IgA deficiency. The study reported a diagnostic sensitivity of 91.2% for tissue tTG‐IgG and 75.8% for EMA‐IgG, while the sensitivity for IgG‐anti‐DGP was found to be 88.2%. Researchers concluded that IgG‐anti‐DGP could be useful for diagnosing CD in patients with IgA deficiency [118]. Prince and colleagues, by assessing the impact of IgA deficiency on the serologic detection of CD, suggested that tTG‐IgG might be the most reliable alternative for identifying CD in patients with IgA deficiency [119].

Despite the important role of IgG‐based tests in detecting CD in IgA‐deficient patients, their effectiveness in assessing disease activity or adherence to a GFD remains controversial. Chow stated that in patients with complete IgA deficiency, IgG serologies may remain elevated even if histologic recovery has occurred. Therefore, relying solely on IgG serologies to evaluate disease activity or adherence to a GFD in IgA‐deficient individuals is unreliable [115]. This finding was also observed in a study by Cataldo and colleagues, who reported that 4 out of 34 IgA‐deficient patients on a strict GFD still had detectable tTG‐IgG antibodies [120]. Korponay‐Szabo and colleagues, in a study involving 325 IgA‐deficient subjects, including 78 with CD, found that the decline in IgG autoantibodies is notably slow in these patients, with most remaining positive even after more than 2–3 years on a GFD. Researchers suggest that the slow decline of IgG celiac antibodies could be linked to the immunoregulatory abnormalities associated with IgA deficiency. Nonetheless, similar to other studies, they conclude that tTG‐IgG and EMA‐IgG tests are highly effective for detecting CD in patients with IgA deficiency [121].

IgM deficiency (SIgMD) is a rare disorder with a prevalence of 1 in 15,000 characterized by reduced levels of IgM with normal levels of other immunoglobulins [122]. This condition has been linked to CD, as illustrated by Montenegro and colleagues, who described the case of a patient who presented with abdominal pain, diarrhea, and weight loss. Laboratory investigations revealed reduced IgM levels and negative results for tTG‐IgA and tTG‐IgG. Histology showed villous atrophy and diffuse immature lymphocytes. tTG mRNA levels in the mucosa were elevated sixfold. Following implementing a GFD, the patient experienced symptom resolution, restoration of villous architecture, and normalization of mucosal tTG mRNA levels to those observed in healthy individuals. After 1 year on the GFD, IgM levels fully returned to normal, and a follow‐up duodenal biopsy showed a reduction in immature lymphocytes with normal mature immune cells. Researchers propose that there could be a specific link between IgM deficiency and seronegative CD. They also suggest that a GFD may help reverse IgM deficiency by enhancing lymphocyte maturation [123]. Additionally, Goldstein and colleagues, in a review of 49 pediatric cases of SIgMD, identified 5 cases associated with CD, where serum IgM levels normalized following adherence to a GFD [124]. Despite observed cases suggesting a potential link between SIgMD and CD, the association remains inadequately explored in the literature. Further research is required to clarify the underlying mechanisms and assess the clinical significance of SIgMD in CD.

Table 1 provides a summary of the changes in serum proteins and their clinical relevance in CD.

**Table 1:** Alteration of serum proteins and their clinical utility in celiac disease.

Protein	Alteration in CD	Probable clinical utility in CD
TTR	Decrease [33]	− Track mucosal recovery in CD patients by serial measurements − May contribute to malnutrition associated with CD
ALB	Decrease [38, 40, 41, 45, 49]	− Monitoring the effectiveness of a GFD − Staging RCD and assessing prognosis
IMA	Increase [50, 51]	− Indirectly measure the presence of oxidative stress and ischemia − Diagnostic and monitoring tool to assess the impact of treatment on active CD
AAT	Decrease [56] Increase [58]	Possible association with CD and AATD
AGP	Increase [58]	Further research is needed
CP	Decrease [66, 67] Normal [68, 69]	Identifying copper deficiency in CD^a^
Hp	Increase [74]	May influence susceptibility to CD and disease progression
Tf	Increase [38, 82, 83, 86]	Diagnosing CD‐related IDA
β2M	Increase [58, 95]	− Monitoring CD activity − Assessing the effectiveness of a GFD
CRP	Increase [20, 102]	Monitoring inflammation and cardiovascular risk in CD patients
Ig	IgA deficiency (10–15 times more common than general population) [113, 114, 119] IgM deficiency (rare) [123, 124]	− IgA measurement to avoid false negative results in IgA‐deficient CD patients − IgG measurement for detecting IgA‐deficient patients with CD − Probable link between IgM deficiency and seronegative CD

a Findings suggest that CP measurement may not consistently serve as a reliable marker for copper deficiency in all cases of CD.

## 12 Conclusion

This review highlights the emerging significance of serum proteins as biomarkers in CD. Findings reveal their potential for monitoring disease activity, assessing nutritional status, and evaluating treatment responses, addressing key limitations in current diagnostic and therapeutic approaches. Acute‐phase proteins and immunoglobulins offer additional layers of insight into CD pathophysiology, while markers like TTR and β2M show promise for noninvasive disease management. By advancing the integration of serum protein analysis into clinical practice, this work paves the way for more precise diagnostics and personalized therapies. Future studies should prioritize validating these biomarkers in broader cohorts and exploring their application in innovative treatment strategies.

## Author Contributions

Conceptualization: Mohammad Rostami‐Nejad. Data curation: Sajjad Bakhtiari and Behrooz Ahmadi. Formal analysis: Sajjad Bakhtiari and Behrooz Ahmadi. Methodology: Sajjad Bakhtiari and Behrooz Ahmadi. Project administration: Mohammad Rostami‐Nejad, Mostafa Rezaei‐Tavirani, and Somayeh Jahani‐Sherafat. Supervision: Mohammad Rostami‐Nejad. Validation: Mohammad Rostami‐Nejad. Visualization: Sajjad Bakhtiari and Behrooz Ahmadi. Writing – original draft: Sajjad Bakhtiari and Behrooz Ahmadi. Writing – review and editing: Mohammad Rostami‐Nejad, Nastaran Asri, and Andrea Masotti.

## Ethics Statement

The authors have nothing to report.

## Consent

The authors have nothing to report.

## Conflicts of Interest

The authors declare no conflicts of interest.

## Data Availability

The authors have nothing to report.
